# Supplementary figures and images for: Dynamics of myogenic differentiation using a novel Myogenin knock-in reporter mouse
Source: Skelet Muscle. 2021 Feb 18;11:5. doi: 10.1186/s13395-021-00260-x (PMC7890983; doi:10.1186/s13395-021-00260-x)

Figure S1

Benavente-Diaz et al.

A

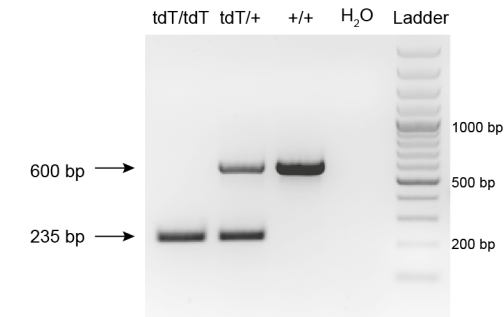

Supplement: Supplementary file 5 — Additional file 5: Figure S1. Genotyping of the ntdTom allele. A. Genotyping of ear-clip samples from MyogntdTom/ntdTom, MyogntdTom/+ and Myog+/+ animals. Myog-ntdTom allele was verified by PCR using primers 16, 17 and 18 (Flp-recombined Myog-ntdTom allele, 236 bp and WT allele 600 bp). [file 13395_2021_260_MOESM5_ESM.pdf]

A

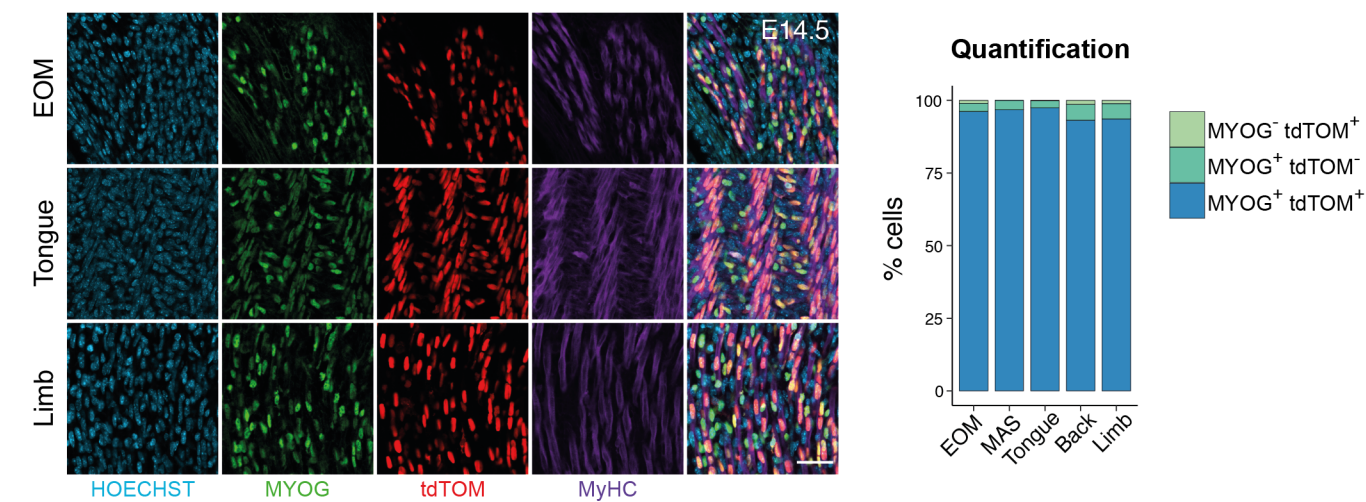

B

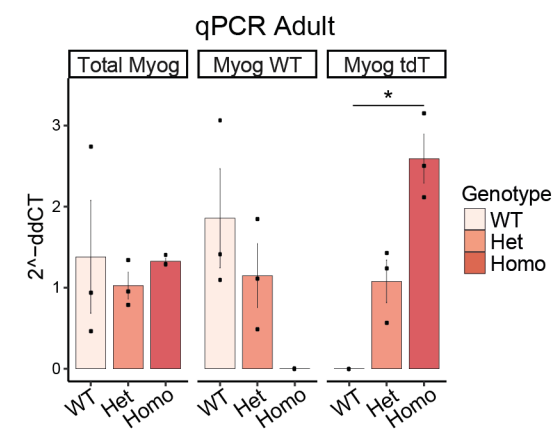

C

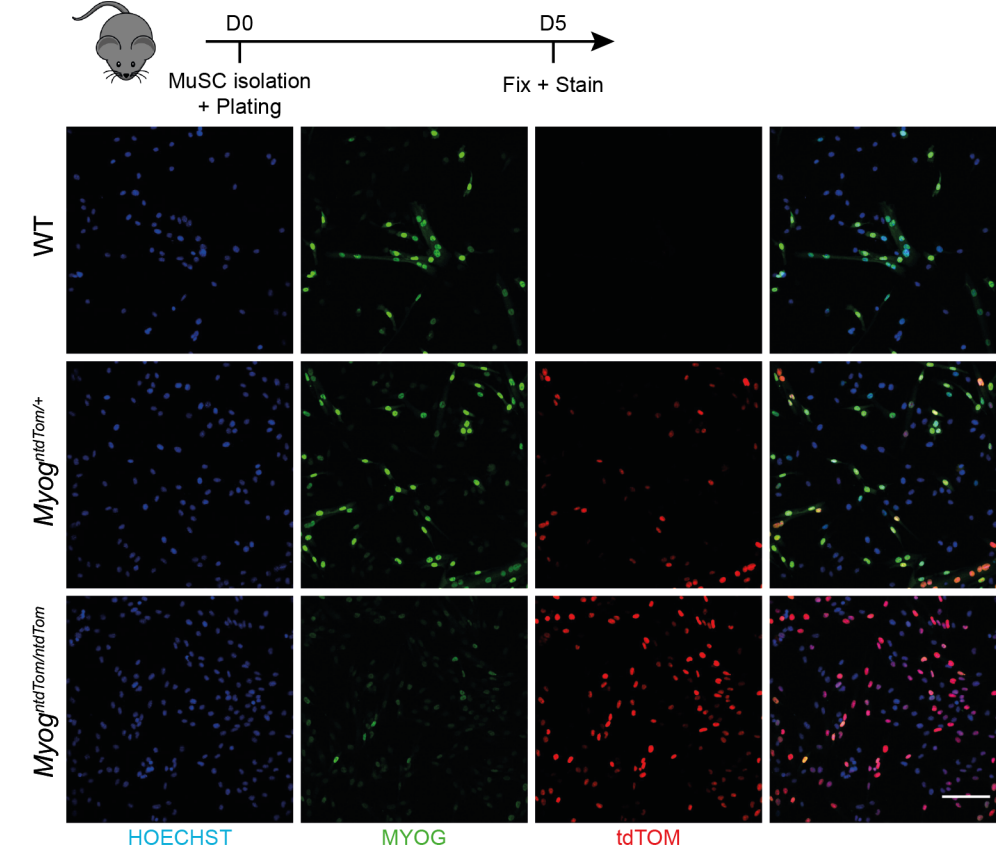

Supplement: Supplementary file 6 — Additional file 6: Figure S2. tdTOM expression recapitulates endogenous Myog expression. A. Immunofluorescence of extraocular (EOM), tongue and limb muscles of MyogntdTom/+ embryo at E14.5. Bar graph shows the quantification of tdTOM and MYOG positive cells. n = 3 embryos, > 100 cells per muscle and per embryo were counted. Scale bar, 40 μm. B. RT-qPCR assessing the levels of total Myog mRNA, the wild-type allele and the tdTom allele specifically from Myog+/+, MyogntdTom/+ and MyogntdTom/ntdTom adult myoblasts using the primer set described in Fig. 1c. n = 3 animals per genotype. Data represents mean ± s.d. Two-tailed unpaired Student’s t-test; * p-value = 0.01 to 0.05. C. MuSCs from limb muscles were isolated from Tg:Pax7-nGFP; Myog+/+, Tg:Pax7-nGFP; MyogntdTom/+ and Tg:Pax7-nGFP; MyogntdTom/ntdTom animals and plated for in vitro differentiation for 5 days. Cells were stained for MYOG and tdTOM proteins. Scale bar, 100 μm. [file 13395_2021_260_MOESM6_ESM.pdf]
