# Supplementary material for: Dynamics of myogenic differentiation using a novel Myogenin knock-in reporter mouse
Source: Skelet Muscle. 2021 Feb 18;11:5. doi: 10.1186/s13395-021-00260-x (PMC7890983; doi:10.1186/s13395-021-00260-x)
Supplement: Supplementary file 7 — Additional file 7: Supplementary Table 1. Primer sequences [file 13395_2021_260_MOESM7_ESM.docx]

**Supplementary Table 1. Primer sequences**

| Primer Number | Primer Name | Sequence |
| --- | --- | --- |
| 1 | Myog_5Fwd | TTAAGG GTCGAC AAAAGTGGCCTAAGAAACCTGCTTTG |
| 2 | Myog_5Rev | CCTTAAGCGGCCGCGTTGGGCATGGTTTCGTCTGG |
| 3 | Myog_3Fwd | TTAAGGTTAATTAAGATTGTCTGTCATACTGGGTGTGC |
| 4 | Myog_3Rev | CCTTAAACTCGAGTTGATAGCAACCAGTCTTTATTCATTTTTT  AAAAA |
| 5 | T2A_NLS_Fwd | TTAAGGGCGGCCGCCGGCAGTGGAGAGGGCAGAGGAAGTCT  TCTAACATGCGGGGACGTGGAGGAAAATCCCGGGCCCCCAA  AAAAGAAGA |
| 6 | NLS_T2A_Rev | GCAGCAAGGTACCGTGTACCTTTCTCTTCTTTTTTGGATCTAC  CTTTCTCTTCTTTTTTGGATCTACCTTTCTCTTCTTTTTTGGGG  GCCCGGGAT |
| 7 | TdT_Fwd | TTGCTGC GGTACC ATGGTGAGCAAGGGCGAG |
| 8 | TdT_Rev | AGTTATGGCCGGCCTCACTTGTAC |
| 9 | FNF_Fwd_3 | GTAGCAAGGCCGGCCACGGTATCGATAAGCTTGATATCGAA  TTCC |
| 10 | FNF_Rev_3 | GCAGCAATTAATTAAACGAAGTTATATTATGTACCTGACTGA  TGAAGTTC |
| 11 | Myog_sgRNA_Fwd | CACCGCCCAACTGAGATTGTCTGTC |
| 12 | Myog_sgRNA_Rev | AAACGACAGACAATCTCAGTTGGGC |
| 13 | Myog_Screen_Fwd | CCCGGAAACAAGCCTGCAG |
| 14 | Myog_Screen_Rev | TGGTGGCTCCCCTCTTGATAATATCAT |
| 15 | TdT_5Rev | TCGCCCTTGCTCACCATGG |
| 16 | Myog-ntdT Fwd | TTCCTGTACGGCATGGACGAG |
| 17 | Myog-ntdT Rev | CAGGACAGCCCCACTTAAAAGC |
| 18 | Myog-ntdT WT | CTTGCTGACCTGAGGGCC |
| 19 | Myog qPCR Total Fwd | TGCCCAGTGAATGCAACTCCC |
| 20 | Myog qPCR Total Rev | CGATGGACGTAAGGGAGTGCA |
| 21 | Myog qPCR WT Fwd | CCCTACAGACGCCCACAATCT |
| 22 | Myog qPCR WT Rev | GGGCTCACATGCACACCCA |
| 23 | Rpl13 qPCR Fwd | GTGGTCCCTGCTGCTCTCAAG |
| 24 | Rpl13 qPCR Rev | CGATAGTGCATCTTGGCCTTTT |
